# Translation into Arabic of: “Changes to publication requirements made at the XVIII International Botanical Congress in Melbourne – what does e-publication mean for you?”. Translated by Ahmed M. Abdel-Azeem and Gihan S. Soliman تغييرات شروط النشر التى اجريت في المؤتمر الدولي الثامن عشر للنبات في لبورن- ماذا يعنى النشر الإلكتروني بالنسبة لك؟

**DOI:** 10.3897/phytokeys.7.2453

**Published:** 2011-11-29

**Authors:** Sandra Knapp, John McNeill, Nicholas J. Turland

**Affiliations:** 1 Department of Botany, The Natural History Museum, Cromwell Road, London SW7 5BD, UK The Natural History Museum London United Kingdom; 2 Royal Botanic Garden, Edinburgh, 20A Inverleith Row, Edinburgh EH3 5LR, UK Royal Botanic Garden Edinburgh United Kingdom; 3 Missouri Botanical Garden, PO Box 299, St Louis, MO 63166-0299, USA Missouri Botanical Garden St. Louis United States of America

## Abstract

يتم تقرير تغييرات في القانون الدولى للتسمية النباتية كل 6 سنوات في أقسام التسميات المرتبطة بالمؤتمرات النباتية الدولية (IBC). وبالمؤتمر الدولي الثامن عشر للنبات الذي عقد في ملبورن ، استراليا ؛ عقد قسم التسميات إجتماعاً خلال الفترة من 18إلى 22 يوليو 2011 وتم قبول قراراتها من قبل المؤتمر في جلسته العامة يوم 30 يوليو. وقد أجريت نتيجة لهذا الاجتماع العديد من التغييرات الهامة على القانون والتي سيكون لها تأثير على نشر أسماء جديدة. وسيسري العمل باثنين من تلك التغييرات في 1 كانون الثاني 2012، وقبل بضعة أشهر من نشر قانون ملبورن. فستصبح المواد الالكترونية التي نشرت على الانترنت على هيئة تنسيق المستندات المحمولة (PDF) مع الرقم التسلسلي المعيارى الدولى (ISSN) أو الرقم المعيارى الدولى للكتاب (ISBN) صالحة للنشر كما سيتم تغيير شرط الحصول على وصف أو تشخيص لاتينى لأسماء الأصنوفات الجديدة إلى شرط الوصف أو التشخيص سواء باللاتينية أو الإنجليزية. بالإضافة إلى ذلك،فإنه بدء من يناير عام 2013 سيكون على الأسماء الجديدة للكائنات الحية التي تعامل كفطريات أن تشمل في البروتولوج (كل ما يرتبط بإسم وبنشره الصحيح) توثيق المعرف صادرا من مستودع معترف به (مثل بنك الفطريات MycoBank) لتصيرصالحة للنشر. ويتم توفير مسودة المواد الجديدة التي تتعامل مع النشر الإلكتروني وتحديد أفضل الممارسات.

ولتشجيع نشر التغييرات التي أدخلت على القانون الدولي للتسمية للطحالب والفطريات ، والنباتات ، سيتم نشر هذه المقالة في المجلات التالية BMC Evolutionary Biology, Botanical Journal of the Linnean Society, Brittonia, Cladistics, MycoKeys, Mycotaxon, New Phytologist, North American Fungi, Novon, Opuscula Philolichenum, PhytoKeys, Phytoneuron, Phytotaxa, Plant Diversity and Resources, Systematic Botany و Taxon.

## المقدمة

في المؤتمر الثامن عشر الدولي للنبات في ملبورن، استراليا، في يوليو 2011 ، تم إعداد تغييرين هامين فى القانون الدولى لتسمية النبات (الآن القانون الدولي لتسمية الطحالب والفطريات والنباتات) وسيكونا ساريان بدءا من 1 يناير 2012 بما سيؤثر على كل من ينشر أسماء تحت حكم هذا القانون .وحيث أن قانون ملبورن سوف لا ينشر حتى منتصف عام 2012 تقريبا، فقد شعرنا أنه سيكون من المفيد توضيح هذه التغييرات، ولا سيما بصلاحية النشر في وسائل الإعلام الإلكترونية (في المواد 29 و 30 و 31). للاطلاع على تقرير موجز عن كل التغييرات على القانون والمقبولة في ملبورن ، انظر ماكنيل وآخرون (2011).

وتم توفير مسودة لصيغة المواد المعدلة والملاحظات والتوصيات المتعلقة بصلاحية النشر لمساعدة المحررين والناشرين لوضع أفضل الممارسات لتنفيذ هذا الجانب من القانون. ونحن نستعرض هنا أيضا ما لا تعنيه تلك التغييرات، من أجل توجيه الراغبين في نشر أسماء جديدة ونماذج بالوسائل الالكترونية. ونحن نحث القراء على الاطلاع على تقرير اللجنة الخاصة المعنية بالنشر الإلكتروني المصاحبة للتغييرات المقترحة قبل المؤتمر (تشابمان وآخرون 2010) ، حيث تم عرض منطق التغييرات التي تم قبولها في القانون الآن. 

## مسودة صياغة المواد المعدلة 29 ، 30 ، و 31 والتوصيات 29A ، 30A ، و31A

نحن هنا نعيد صياغة كلمات جميع المواد المتصلة والملاحظات والتوصيات (مع حذف الأمثلة)، مع التأكيد على التغييرات بكتابتها بخط سميك والصياغة هنا مؤقتة لحين اجتماع لجنة التحرير في ديسمبر 2011 لوضع اللمسات الأخيرة على النسخة المطبوعة من قانون ملبورن.

### المادة 29

29.1. النشر يصبح صالحا، بموجب هذا القانون ، من خلال توزيع المواد المطبوعة (من خلال البيع والتبادل أوالهبة) لعامة الناس أو على الأقل للمؤسسات النباتية ذات المكتبات والتي فى متناول علماء النبات عموما. **والنشر يعد صالحاً أيضا بتوزيع المواد إلكترونيا بتنسيق المستندات المحمولة (PDF ، وانظر أيضا المادة 29.3 والتوصية 29A.1) في منشور على الانترنت مع الرقم التسلسلي المعيارى الدولى (ISSN) أو الرقم المعيارى الدولى للكتاب (ISBN). **لا يعد النشر صالحاً بنشر أسماء جديدة من خلال تبليغها في جلسة علنية أوعن طريق وضع الأسماء في مجموعات أو حدائق مفتوحة للجمهور أو من خلال إصدار الميكروفيلم المصنوع من مخطوطات، أوراق مطبوعة، أو مواد أخرى غير منشورة، أو عن طريق التوزيع الإلكترونى غير المذكور أعلاه.

**29.2. لغرض هذا المقال يعرف «الإلكتروني» بأنه مايمكن الوصول إليه إلكترونيا عبر الشبكة العنكبوتية العالمية.**

***29.3.* في حال استبدال نسق المستندات المحمولة (PDF)، فيقبل خلفا له تنسيق المعايير الدولية التي تحددها اللجنة العامة (انظر الجزء الثالث)**

***29.4.* الصالح ذاته وأي تصحيحات أو تعديلات يجب أن تنشر منفصلة لتعتبر منشورة نشراً صالحاً.**

### توصية 29A

[التوصية الحالية يستعاض عنها بما يلي :]

***29A.1.* 29A.1. ينبغي أن يكون النشر الإلكتروني في نسق المستندات المحمولة (PDF) متوافقا مع نسق المستندات المحمولة/ المحفوظات القياسية ( ايزو 19005).**

***29A.2.* يفضل أن يقوم الناشر بالنشر في المنشورات التي يتم حفظها، وتلبي المعايير التالية بقدر إمكانية التطبيق (انظر أيضا التوصيةA. 1 29) :**

**(*a*) ينبغي أن توضع المواد في مستودعات رقمية عديدة بالانترنت موثوق بها ، مثل مستودع حائزة على شهادة الايزو؛**

**(*b*) ينبغي أن تكون المستودعات الرقمية في أكثر من منطقة واحدة فى العالم ، ويفضل أن تكون في مختلف القارات؛**

**(*c*) تخزن النسخ المطبوعة في المكتبات في أكثر من منطقة واحدة من العالم ، ويفضل أيضاً في قارات مختلفة.**

### المادة 30

***30.1.* لا يشكل المنشور إلكترونيا منشورا صالحاُ قبل 1 يناير 2012.**

***30.2.* لا يعد المنشور الكترونيا نشرا صالحا إذا كانت هناك أدلة مرتبطة بالمنشور أو داخله على انه مجرد نسخة أولية كانت ستستبدل أو سيتم استبدالها بنسخة يعتبرها الناشر نهائية وفي تلك الحالة تكون النسخة النهائية تلك فقط هي الصالحة للنشر.**

*30.3.* يعتبر النشر عن طريق مخطوطات لاتمحى قبل 1 يناير 1953 نشرا صالحا. أما المخطوطات التى لا تمحى والتي انتجت في وقت لاحق لا تعد نشرا صالحا.

*30.4.* هدف هذه المادة، ان المخطوطة التى لاتمحى عبارة عن مادة مكتوبة بخط اليد وتم انتاجها عن طريق عملية ميكانيكية أو طباعة (مثل الطباعة الحجرية ، الاوفست، أو النقش معدني).

*30.5.* النشر في أو بعد 1 يناير 1953 في كتالوجات التجارة أو الصحف غير العلمية، وفى أو بعد 1 يناير 1973 في قوائم تبادل البذور، لا يشكل نشرا صالحا.

*30.6.* توزيع المطبوعات فى أو بعد 1 يناير 1953 المصاحبة للعينات لا يشكل نشر صالحا. .

*Note 1.* أما إن تم توزيع المادة المطبوعة مستقلة عن العينات، يعد النشر صالحا.

*30.7.* النشر في أو بعد 1 يناير 1953 لعمل مستقل بدون رقم مسلسل على أنها أطروحة قدمت إلى جامعة أو معهد تعليمي أخر بغرض الحصول على درجة لا يعد نشرا صالحا ما لم يتضمن بيانا واضحا (في إشارة إلى شروط قانون النشر الصالح) أو أدلة داخلية أخرى على أنها تعتبر نشرا صالحا من قبل مؤلفها أو ناشرها

*Note 2.* ويعتبر وجود الرقم المعيارى الدولى للكتاب (ISBN) أو بيانا باسم المطبعة، أو الناشر أو الموزع في النسخة الأصلية المطبوعة دليلا داخليا أن العمل كان مخصصا للنشر نشرا صالحا.

### توصية 30A

***30A.1.* ينبغي الإشارة بوضوح إلى أن الإصدار الأولي والنهائية لذات النشر الالكتروني كانتا كذلك عند إصدارهما للمرة الأولى.**

*30A.2.* ينصح وبشدة أن يتجنب المؤلفين نشر أسماء وأوصاف جديدة أو تشخيص لأصنوفات جديدة (تسميات حديثة) في أي مطبوعات سريعة الزوال من أي نوع ، ولا سيما المواد المطبوعة التي أنتجت في أعداد محدودة وغير مؤكدة ، والتي عندها تكون ديمومة النص محدودة ، وبالتالي فإن النشر الصالح بالرجوع إلى عدد النسخ يصبح غير واضح ، أو أنه من غير المرجح أن يصل إلى عامة الناس. وينبغي أيضا أن يتجنب المؤلفين نشر أسماء وأوصاف جديدة أو تشخيصات في الدوريات الشعبية، في مجلات للملخصات، أو على قصاصات تصويب

*30A.3.* ولتسهيل الإتاحية عبر الزمان والمكان ، يجب على المؤلفين ممن يقوموا بنشر أسماء جديدة إعطاء الأفضلية للدوريات التي تنشر المقالات التصنيفية بانتظام. **و إلا فعليهم إرسال نسخة من المنشور (سواء كان منشورا مطبوعا أو إلكترونيا) إلى مركزا للفهرسة مناسباً للمجموعة التصنيفية ، أما المنشورات التي لا توجد إلا على هيئة مطبوعة** sفينبغي إيداعها في بما لا يقل عن عشرة ، ولكن يفضل أكثر ، مكتبات نباتية أو غيرها من المكتبات المتاحة عموما في جميع أنحاء العالم

*30A.4.* وينصح أن يقوم الكتاب والمحررين بذكر الأسماء الجديدة في الملخصات والمختصرات، أو حصرها في فهرس في المطبوعة.

### المادة 31

*31.1.* تاريخ النشر الصالح هو التاريخ الذي تصبح فيه المادة المطبوعة **و الإلكترونية** متاحة كما تم تعريفها فى المادة 29 و 30. في غياب بروفة طباعة لإثبات تاريخ آخر، فإنه يجب قبول التاريخ الظاهر على المواد المطبوعة **أو الإلكترونية** على انه صحيحا.

[ملاحظة 1 القائمة يستعاض عنها بما يلي :]

***31.2.*عندما يتم إصدار منشور إصداراُ الكترونيا ومطبوعا بالتوازي فيجب معاملة كلاهما كنشرا صالحا في ذات التاريخ ما لم يكن تاريخ الإصدار مختلفا طبقاً للمادة 31.1.**

*31.3.* عندما تطرح بعض المستلات من دوريات أو من أعمال أخرى للبيع مقدما ، فإن التاريخ الموجود على المستلة يقبل على أنه تاريخ النشر الصالح ما لم يتوفر دليل على خطأ ذلك.

### توصية 31A

*31A.1.* ينبغي قبول التاريخ الذي قام فيه الناشر أو وكيل الناشر بتوفير المواد المطبوعة إلى واحدة من شركات الطيران المعتادة للتوزيع على الجمهور على أنه تاريخ النشر الصالح

## أفضل الممارسات

على مؤلفي الأسماء الجديدة ، والمحررين والناشرين جميعا الاهتمام بالتأكد أن المنشورات والتي تشتمل على أسماء جديدة توافق جميعها قانون ملبورن ، بحيث يتم نشر الأسماء فيه بشكل صالح. ونقترح أن من يقومون بالنشر في المجلات أو سلسلة من الدراسات والكتب التي لها طبعات على الانترنت التواصل مع المحررين بحيث يمكن التوصل لأفضل الممارسات في جميع أنحاء المجتمع بأسرع وقت ممكن. وقد قام العديد من الناشرين بتناول المسائل التي تتصل بالنشر الإلكتروني للمستجدات لبعض الوقت (انظر ناب ورايت 2010 ؛ المبادئ التوجيهية PLoS One [http://www.plosone.org/static/policies.action#taxon وقد بدا منهم اهتماما واضحا بسريان التغييرات الجديدة في القانون بفاعلية.

ومن الممارسات التي نراى أنها ستساعد في المراحل الأولية للنشر الإلكتروني للمستجدات وفقا لقانون ملبورن هي ::

• أن يحمل كل مقال تاريخ نشره بصورة واضحة (كما هو الحال في العديد من المجلات ، على سبيل المثال New Phytologist أو Nature).
• إذا تم إصدار نسخة على الإنترنت في وقت سابق ليست هي نفس الصيغة النهائية (وبالتالي لايعد هذا مكان النشر الصالح) ، يختم كل مقال بهذه الحقيقة بوضوح (على سبيل المثال American Journal of Botany).
• عرض الرقم التسلسلي المعياري الدولي أو الرقم المعياري الدولي للكتاب المنشور بوضوح على كل مادة لتساعد المفهرسين لإنشاء نشر صالح.
• النشر في مجلات (أو سلسلة من الدراسات) تشارك في نظام CLOCKSS (انظر ناب ورايت 2010 للحصول على وصف) أو أرشيف دولي آخر ونظام حفظ يضمن الحفظ على المدى الطويل.
• وينبغي أن ينبه المؤلفين للأسماء الجديدة بالوسائل الالكترونية مركز الفهرسة المناسب لهم على النحو الموصى به في التوصية 30 A.3 . – مما سيساعد المفهرسين الذين قد لا يكونوا على وعي بالأسماء المنشورة إلكترونيا ان لم يتم تنبيههم بتلك الكيفية.

## ما *لا* تعنيه هذه التغييرات 

على الرغم من أن المواد الجديدة والتوصيات تستخدم مصطلحي نسق المستندات المحمولة ونسق المستندات المحمولة/ المحفوظات القياسية PDF و PDF / A ، فإن هذا لا يعني حتمية أن تصدر المنشورات حصريا في هذا الشكل لتكون صالحة للنشر. على سبيل المثال ، بعض أوراق المجلات على الإنترنت تصدر في تنسيق لغة توصيف النص التشعبي (HTML) بالتوازي مع نسق المستندات المحمولة. وفي مثل هذه الحالات ، سيتم نشر نسخة نسق المستندات المحمولة نشرا صالحا. أن شرط أن اللجنة العامة للتسميات النباتية سوف تنقل قبول صيغة معيار دولي جديدة ، في حال استبدال نسق المستندات المحمولة طالما يعني أن مؤلفي الأسماء الجديدة والمجتمع ممن يستخدموا القانون يستطيعون البقاء على علم بالتقدم في المجال وأن القانون سوف يكون محميا من التقادم.

ستخدام الوسائل التالية للنشر الإلكتروني لن تؤدي للنشر الصالح للمستجدات بموجب قانون ملبورن :

• النشر على مواقع الانترنت أو في وثائق زائلة متاحة عبر شبكة الإنترنت (هناك معايير صارمة لمنح الأرقام التسلسلية المعيارية الدولية [http://www.issn.org]).
• النشر في مجلات دون رقم تسلسلي معياري دولي مسجل أو رقم تسلسلي معياري دولي الكتروني.
• النشر في كتب دون الرقم المعياري الدولي للكتاب أو الرقم المعياري الدولي للكتاب الالكتروني.

إن التوصية المعتمدة كمشورة لحفظ نسخة مطبوعة من أي نشر إلكتروني في مكتبة تقترح لعلماء النبات عملا إجرائيا، إلا أنها لا تنص على ممارسة معيارية أو بروتوكول يتبعه أمناء المكتبات. فأمناء المكتبات أنفسهم في مرحلة انتقالية معقدة بين طرق النشر (جونسون ولوثر 2007) ، وقد يجد علماء النبات أن أمناء المكتبات غير راغبين أو غير قادرين على استيعاب إحدى المطبوعات الورقية كمنشور مفرد في حال كونه كبير الحجم.

## تغييران آخران هامان في *القانون* يتعلقان بنشر الأسماء

التغيير الثاني في القانون الذي تم اعتماده في ملبورن على أن يسري بدئه من يناير 2012 هو أن الوصف أو التشخيص المطلوب للنشر الصالح لأسم جديد لجميع الكائنات التي ينطبق عليها القانون يمكن أن تكون بالإنجليزية أو اللاتينية وهذا هو الشرط الحالي لأسماء حفريات النبات إلا أن كل الأسماء الغير حفرية الجديدة مازالت تتطلب وصفا لاتينيا أو تشخيصا (الفطريات والنباتات من 1 يناير 1958 والطحالب بما يشمل البكتيريا الخضراء المزرقة لو تمت معاملتها بموجب القانون من يناير 1958). وهذا لا يطبق فى حالة الأسماء العلمية والتي ستستمر لاتينية أو تعامل كلاتيني. والمتطلبات الفردية لمجلة باللاتيني و/ أو الإنجليزي يحددها محرري تلك المجلات.

والتغيير الثالث للقانون المعتمد في ملبورن يرتبط بنشر الأسماء لكنه لا يسري حتى 1 يناير 2013 (ليس يناير 2012 كما نقله ميلر وآخرون 2011) وهو أن الأسماء الجديدة للكائنات التي تعامل كفطريات يجب أن، بالإضافة إلى شرط النشر الصالح ، تشتمل في البروتولوج (كل ما يرتبط بالإسم وصلاحية نشره) على المصدر الذي يحدد المعرف وصادر من مستودع معترف به (مثل بنك الفطريات ]http://www.mycobank.org/[وينشر ذلك بشكل منفصل. إن شروط المعرف الفريد لأسم جديد من الفطريات بحلول 1 يناير 2013 أو بعد ذلك لا ينطبق على النباتات أو الطحالب ولا حاجة لمؤلفي أسماء جديدة في هذه المجموعات أن يطلبوا معرفي علوم حياة (LSIDs) أو أي معرفين آخرين من مراكز الفهرسة.
